# Prevalence of Disordered Eating Among International Sport Lead Rock Climbers

**DOI:** 10.3389/fspor.2020.00086

**Published:** 2020-07-24

**Authors:** Lanae M. Joubert, Gina Blunt Gonzalez, Abigail J. Larson

**Affiliations:** ^1^Exercise Science Laboratory, School of Health and Human Performance, Northern Michigan University, Marquette, MI, United States; ^2^Exercise Science Laboratory, Department of Kinesiology, Health and Imaging Sciences, Morehead State University, Morehead, KY, United States; ^3^Exercise Science Laboratory, Department of Kinesiology and Outdoor Recreation, Southern Utah University, Cedar City, UT, United States

**Keywords:** rock climbing, eating attitudes, eating behavior, sport performance, sex differences, nutrition

## Abstract

Disordered eating (DE) is characterized as a range of irregular eating patterns or behaviors, which may lead to pathological eating or a clinical eating disorder diagnosis. DE patterns are associated with a variety of negative health outcomes. The prevalence of DE is highest in female athletes who participate in aesthetic or weight dependent sports. Elite rock climbers tend to be strong, small and lean, but the prevalence of DE in rock climbers is unknown. The purpose of the present study was to assess DE prevalence in a large group of international rock climbers and to explore the relationship between sport rock climbing ability and DE. A web-based survey assessed both DE (Eating Attitudes Test-26) and climbing ability based on the International Rock Climbing Research Association's position statement on comparative grading scales. The survey was distributed to international climbing communities; 810 individuals attempted the survey; 604 completed all questions; 498 identified as sport lead climbers. The majority of sport lead climbers were lower grade/intermediate (57.8%), compared to advanced (30.7%) and elite/higher elite (11.4%), and male (76.9%). Forty-three sport lead climbers reported a score of 20 or above on the EAT-26 indicating an 8.6% prevalence of DE in this sample. Male climbers had a DE prevalence of 6.3% (24 of 383) and female climbers more than doubled that with 16.5% (19 of 115). Chi-square analysis revealed that DE was associated with climbing ability level [χ2 (2, *n* = 498, 8.076, *p* = 0.02)], and when analyzed by sex, only the female climbers had a significant relationship of DE with climbing ability [χ2 (2, *n* = 115, 15.640, *p* = 0.00)]. These findings suggest sport lead rock climbers are not immune to DE and that the risk is elevated in female climbers, particularly at the elite/high elite climbing ability level. Our research indicates further investigations are warranted to determine if and how disordered eating behaviors affect health and performance of adult rock climbers.

## Introduction

Early research by Black and Burckes-Miller (1988) postulated that, due to the unique pressures associated with sport participation (e.g., athletic performances, coaches' expectations, sporting environment, and subculture), athletes may be more prone to engage in disordered eating (DE) and unwise weight management techniques, which may lead to eating disorders (EDs). Nearly 15 years later, research by Sundgot-Borgen and Torstveit (2004) provided evidence supporting these hypotheses. In their large, well-conducted study among Norwegian elite athletes (*n* = 1,259) and controls (*n* = 1,203), they concluded that the prevalence of EDs was higher in elite athletes (13.5%) compared to controls (4.6%). In addition, EDs were higher in female athletes (20.1%) than in male athletes (7.7%). Incidence of EDs was also found to vary between sporting categories; among female athletes, those who competed in aesthetic sports (e.g., gymnastics) had the highest incidence (42%), while among male athletes those who competed in antigravitational sports (e.g., high jump, long jump, and triple jump) had the highest incidence (22%). Athletes who competed in technical and ball game sports had a lower incidence of EDs when compared to athletes of the same sex who competed in leanness-dependent and weight-dependent sports; however, incidence was still higher than in the general population. Given these figures and the consequences of DE and EDs on physical and mental health as well as athletic performance (Kärkkäinen et al., 2018), quantifying ED risk in less-studied athletic populations is necessary in order to identify individuals at high risk and implement appropriate prevention and intervention strategies.

Rock, or sport, climbers represent a population of athletes who may be at increased risk of developing EDs, albeit little research has examined ED risk in climbers. Competitive rock climbing requires intense kinesthetic awareness of the body and its movement as well as an exceptional strength to bodyweight ratio. As such, good climbers tend to have a lean build and low body mass (Watts et al., 1993, 2003; Novoa-Vignau et al., 2017). Climbing can be considered an antigravitational sport and success is supported by a high strength-to-mass ratio, which diet may strongly influence (Tayne et al., 2019). Although research regarding the dietary intake patterns of climbers is scarce (Zapf et al., 2001; Merrells et al., 2008; Krzysztof and Judyta, 2019), there is anecdotal evidence and published personal testimony of climbers practicing DE behaviors in order to minimize body weight and thus potentially enhance performance (Taylor and Geldard, 2008; Samet, 2013). Because of the myriad of physical and mental consequences associated with long term energy deficiency, and the prevalence of “the lighter the better” mentality among some climbers, the medical community has shared concerns regarding the likely inadequate macronutrient intake and potential for EDs among many climbers (Lutter et al., 2017). On this basis, the Austrian Climbing Organization uses Body Mass Index (BMI) values to help determine eligibility for competing in an effort to prevent disordered eating, and concern has been raised regarding the risk of adolescent climbers developing anorexia athletica.

Despite these worrisome ED indicators, to date, only one peer reviewed study has assessed ED prevalence among rock climbers (Michael et al., 2019). This study examined the dietary habits and eating attitudes of a small sample (13 males, 9 females), of adolescent (14.2 ± 1.9 years) competitive climbers who ranged from intermediate to advanced climbing ability. Results from the 3-day dietary recall and Eating Attitudes Test-26 (EAT-26) indicated the majority (82%) of climbers did not meet their target energy intake (target = 2,471 ± 493 kcal·day−1; actual = 1,963 ± 581 kcal·day−1) (*p* = 0.01) but average EAT-26 scores were 5.3 ± 4.1, indicating minimal risk of DE attitudes/behaviors. Additionally, there were no associations between energy intake and EAT-26 score (*R*^2^ = 0.245, *p* = 0.27) or climbing ability and EAT-26 score (*R*^2^ = 0.274, *p* = 0.23). These data suggest young, adolescent climbers fail to meet energy needs but exhibit minimal risk of DE. However, these findings have not yet been replicated in older adolescents or an adult population of climbers. It is possible that eating attitudes change and risk of developing an ED increases with maturation and typical pubatorial increases in body weight and/or with continued exposure to competitive climbing culture. Therefore, larger studies with more diverse samples are needed to quantify risk in the larger climbing population.

The identification of individuals with DE behaviors can be accomplished via several screening tools. The EAT-26, a 26-item inventory, developed by Garner et al. (1982), has been used to assess a range of attitudes related to eating behavior in athletes and non-athletes. A score of 20 and above is established as a cut-off value to identify individuals with possible DE behavior. The EAT-26 has several advantages when assessing EDs in male and female, athletic populations. Namely the EAT-26 is thought to better predict ED pathology in males compared to other surveys, such as the Eating Disorder Inventory, because it evaluates food preoccupation rather than drive for thinness (Gleaves et al., 2014). Although not specifically developed for use in athletes, the EAT-26 has been frequently used to identify EDs in a variety of athletic populations, thereby making it a useful tool to compare ED prevalence rates between sports, sexes, and ability levels. The EAT-26 has been shown to have an accuracy rate of at least 90% when used to differentially diagnose those with and without ED, according to Mintz and O'Halloran (2000). As such, the EAT-26 was used in the present study in order to evaluate and compare the prevalence of DE in male and female sport lead rock climbers with varying climbing abilities.

## Purpose

The primary purpose of this study was to assess DE prevalence among a large international, heterogeneous sample of sport lead rock climbers with varying abilities. Secondarily, we wanted to determine if there was a relationship between prevalence of DE and climbing ability and if incidence of DE differed between male and female climbers. Based on previous research, we hypothesized that climbers would have a higher prevalence of disordered eating compared to previously reported values in the general population. We further hypothesized that prevalence of DE would be higher in women than men and similar to those found in aesthetic and antigravitational sport populations, respectively.

## Materials and Methods

### Participants

We aimed to recruit an international sample of 500 adults that were currently actively participating in rock climbing. Participants were recruited via email through the International Rock Climbing Research Association (IRCRA) delegation and were provided a link to the electronic survey. The authors also dispersed the survey within their own rock climbing communities within the U.S. using email and social media. It was open to collect responses for 3 months during late summer and early fall of 2017.

Eight hundred and ten individuals attempted the survey, 604 had complete responses to all questions, and 498 self-identified primarily or secondarily as sport lead climbers. Sport lead climbing was defined as a type of lead climbing where the anchoring system is fixed into the rock (usually a steel bolt) and the climber attaches quickdraws (webbing material with 2 carabiners) to the bolts as the climber ascends. The climbing rope is tied to the climber's harness, threaded through a carabiner on the quick draw that gets clipped to the fixed anchor, and connected to a belayer (partner) or an auto-belay device. These 498 sport lead climbers were analyzed in this study.

### Procedures

This study received ethical approval from the Internal Review Board of Northern Michigan University (#HS17-869). After reading the first page explaining the purpose of the research and the types of questions to expect on the survey, participants used a checkbox on the questionnaire to indicate their informed consent in accordance with the Declaration of Helsinki. All surveys were completed anonymously. Only individuals 18 years and older who completed all questions and self-identified as participating in sport lead climbing were included in the present analysis.

### Electronic Survey Instrument

The survey instrument was developed and pilot tested by 7 advanced level climbers (IRCRA mean climbing ability level of 18) with ample rock climbing research experience. Their feedback was utilized to reword questions and enhance the electronic questionnaire experience for mobile devices. Qualtrics software (version 2017; Qualtrics, Provo, UT) was used to develop the web-based electronic survey tool and to collect participant responses. There were 42 total questions in 3 main sections.

Section 1 included basic demographics including self-reported age, biological sex, height, weight, body composition, and country of residence. BMI was calculated from height and weight.

Section 2 focused on climbing characteristics. Participants were required to check the types of climbing they had participated in within the past 365 days from the following 6 choices: bouldering, top-roping, traditional lead (placing gear), sport lead (clipping bolts), free solo, and speed climbing. They were asked about the types of climbing they devoted the most time doing in the past 3 months. Those that selected sport lead climbing as a primary or secondary choice were included in the present analysis. Sport lead climbing ability was self-reported based on the IRCRAs recommended standards for reporting a rock climber's ability in a universal format (Draper et al., 2016). Their sport lead climbing ability was selected from a drop down menu of IRCRA climbing ability levels (1–32) that most closely matched their abilities according to their current best redpoint, which was defined as completing a clean ascent (no falls or weighting the rope) after having one or more practice attempts. For example, an IRCRA level of 15 would equate to a 5.11b on the Yosemite Decimal System scale or a 6c in the French Sport scale and be considered an intermediate level for males and advanced level for females.

Section 3 included the EAT-26 with 26 questions and was used with permission (Garner et al., 1982). A tallied score ≥ 20 on the EAT-26 was considered indicative of DE behavior (Garner et al., 1982). In addition, the very last question on our survey specifically asked if the participant had been treated for an ED any time in their life with a yes or no response option.

### Data Analysis

All data analyses were conducted using the Statistical Package for Social Sciences (SPSS, version 25.0, IBM Corp. Released 2017, Armonk, NY: IBM Corp.) with a significance level set at *p* < 0.05. BMI was calculated from self-reported heights and weights in kg/m^2^. The EAT-26 question responses were scored and tallied in accordance with its rating scale (Garner et al., 1982) and grouped as DE (>20 on EAT-26) or NO DE (<20 on EAT-26). Percentages and mean ± SD are reported for descriptive data. The prevalence of DE was reported in percentages of the total sample, by sex, and by climbing ability level. A Pearson correlation coefficient was used to determine the relationship between BMI and climbing ability in males and females. To discern if DE was associated with climbing ability, we grouped levels 1+2 and levels 4+5 and performed Chi-square analyses with 3 climbing ability groups (low, medium, high) for males and females.

## Results

A total of 810 individuals attempted the survey, however only 604 had complete answers for all questions. Of the 604 completed surveys, the highest percentage of responders identified themselves as sport lead climbers either as their primary or secondary form of climbing (82%; *n* = 498) and these participants were included in the present analyses. These rock climbers represented 33 countries, with the top three highest frequencies from the United States (63%), Canada (8%), and Spain (6%) and the majority were male (77%; *n* = 383). Mean age of the sample was 32 ± 9 years.

Self-reported sport lead climbing abilities ranged from 3 to 31 on the IRCRA scale. Grouped by climbing abilities and sex for the 5 ability levels (lower grade, intermediate, advanced, elite, higher elite), the highest percentage of climbers that completed the survey were considered intermediate for both adult males *(*climb ability of 10–17; *n* = 196; 51.2%) and females (climb ability of 10–14; *n* = 54; 46.1%).

BMI and age for each sex was organized by climbing ability level, see Table 1. The overall average BMI was 22.7 ± 2.6 kg/m^2^ and average age was 32 ± 9 years. There was a general trend of better climbers having a lower BMI for both the female (*r* = −0.329, *p* = 0.00) and male sport lead climbers (*r* = −0.237, *p* = 0.00).

**Table 1:** Sport lead rock climbers' BMI and age by climbing ability level.

	**Males (*****n*** **=** **383)**			**Females (*****n*** **=** **115)**		
	**Total sample (%)**	**BMI (kg/m^**2**^)**	**Age (yrs)**	**Total sample (%)**	**BMI (kg/m^**2**^)**	**Age (yrs)**
Total sample	76.9	22.9 ± 2.6	32 ± 9	23.1	21.9 ± 2.5	33 ± 9
*Lower grade*	7.8	24.9 ± 2.7	33 ± 11	15.7	23.0 ± 3.0	30 ± 8
*Intermediate*	51.2	23.0 ± 2.7	30 ± 8	46.1	22.2 ± 2.4	32 ± 10
*Advanced*	30.7	22.7 ± 2.4	33 ± 10	20.0	21.6 ± 2.4	32 ± 8
*Elite*	10.0	21.5 ± 1.2	35 ± 7	17.4	20.6 ± 2.0	36 ± 8
*Higher elite*	1.4	23.3 ± 2.1	37 ± 5	0.8	19.9	44

*Data expressed in percentages of total sample for each climbing ability with BMI and age expressed as mean ± SD within sex*.

*BMI—Body Mass Index*.

*IRCRA level—International Rock Climbing Research Association's climbing ability scale with 5 levels of climbing abilities; lower grade, intermediate, advanced, elite, higher elite (Draper et al., 2016)*.

Table 2 expresses DE prevalence for each climbing ability grouping and between sexes. Using the pre-established cutoff score of >20 on the EAT-26 questionnaire as identifying individuals at risk for or with DE, we found 43 of the 498 sport lead climbers with this score, indicating an 8.6% overall DE prevalence. Additionally, 21 of 498 (4.2%) reported to have been treated for an eating disorder.

**Table 2:** Sport lead rock climbers' DE by climbing ability.

**Males**		**Females**	
**Sport Lead ClimbAbility Level(*n*)**	**Sport Lead Climbers with DE *n* (%)**	**Sport Lead ClimbAbility Level(*n*)**	**Sport Lead Climbers with DE *n* (%)**
Lower grade (21)	3 (14.3)	lower grade (18)	1 (5.5)
Intermediate (196)	10 (5.1)	intermediate (54)	4 (7.4)
Advanced(130)	10 (7.7)	advanced (23)	5 (21.7)
Elite(30)	1 (3.3)	elite (20)	9 (45)
Higher elite (6)	0 (0)	higher elite (1)	0 (0)
Total DE	24 (6.3)	Total DE	19 (16.5)
Total treated for ED	7 (0.2)	Total treated for ED	14 (12.2)

*Data expressed in absolute numbers (n) and percentages (%) within each climbing ability level*.

*International Rock Climbing Research Association's climbing ability scale with 5 levels of climbing abilities; lower grade, intermediate, advanced, elite, higher elite (Draper et al., 2016)*.

*DE—disordered eating determined by scoring 20 or above on EAT-26*.

*ED—selecting yes to the question “at any time in your life have you been treated for an eating disorder?”*.

Among all of the male sport climbers, DE prevalence was 6.3% (24 of 383). Three of the 21 males with lower grade climbing abilities had DE (14.3%). However, a much higher prevalence was seen in the female climbers with an overall DE prevalence of 16.5% (19 of 115) and nearly half of the females (9 of 21) in the highest two climbing ability levels (elite and high elite) had DE (42.9%).

The final Chi-square analysis reported the overall sample as χ^2^ (2, *n* = 498, 8.076, *p* = 0.02), suggesting DE was associated with climbing ability levels. For males, the χ^2^ (2, *n* = 383, 1.224, *p* = 0.54) revealed no significant associations between DE and climbing ability. However, for the females, the χ^2^ (2, *n* = 115, 15.640, *p* = 0.00) suggested that DE was indeed associated with climbing ability.

## Discussion

This study is the first to publish prevalence data of DE in a large, international, sample of adult rock climbers with a variety of climbing abilities. Using the EAT-26, we found an 8.6% prevalence of DE in our sample of 498 sport lead rock climbers, which is similar to what is reported for other athletic groups. For example, in a large Norweigan cohort (*n* = 3,000), subclinical or clinical EDs were found, also using the EAT-26, in 13.5% of elite athletes compared to 4.6% of the non-athlete controls (*p* < 0.001) (Sundgot-Borgen and Torstveit, 2004). We acknowledge that the EAT-26 was originally intended to detect DE in the general population and therefore may not accurately represent DE prevalence in athletic populations (Pope et al., 2015). As such, and suggested by some researchers attempting to examine disordered eating behaviors in athletic populations (Beals and Manore, 1994; Smolack et al., 2000; Byrne and McLean, 2001; Sundgot-Borgen and Torstveit, 2004; Reinking and Alexander, 2005; Pope et al., 2015), clinical interviews and validated tools specifically designed for active populations are needed to obtain more accurate DE prevalence data, especially in male athletes.

In the present study, and as expected, the prevalence of DE was higher among female climbers compared to male climbers (16.5 and 6.3%, respectively). Previous research also reports higher incidence of DE among female athletes compared to male athletes with the highest prevalence among elite female athletes competing in aesthetic sports such as gymnastics and figure skating (42%) (Sundgot-Borgen and Torstveit, 2004). We found similar DE outcomes in our sample of elite female climbers (43%). Additionally, our sample of female sport lead rock climbers of all abilities had DE prevalence rates (16.5%) comparable to those reported of female gymnasts and swimmers (16%) (Anderson and Petrie, 2012). Contrary to our hypothesis, our sample of elite male climbers had a lower incidence of DE (2.7%) compared to previously reported values in elite male athletes who participated in antigravitation sports (22%), ball game sports (5%) and endurance sports (9%) (Sundgot-Borgen and Torstveit, 2004).

As very few high level female sport lead climbers responded to our survey (*n* = 21), it is unclear whether this is a represent ative sample of the number of females climbing at this skill level. Currently there is no known international database of climber demographics and skill level. However, the lack of participants in the elite and higher elite categories is consistent with other survey-based climbing studies and is a limitation of the current study as well as the existing body of research (Gonzalez, 2019; Grønhaug, 2019). Despite the small size of this subsample, the incidence of DE among the elite female climbers who responded to our questionnaire is striking and warrants additional research. Symptoms and potential drivers of EDs in female athletes include body dissatisfaction and exposure to high standards (i.e., appearing very lean), self-imposed expectations of athletic perfection, and a belief in the inverse relationship between body size and performance (Sanborn et al., 2000). Furthermore, constant evaluation of physical appearance by coaches and peers can lead to negative body image if these standards are not met (de Bruin et al., 2011). Although the etiology of EDs in climbers has not yet been explored, evaluating constructs shown to be salient mediators and moderators of EDs in other athletic populations seems to be a reasonable place to start. Hopefully future research will allow us to better understand drivers and risk factors of DE in climbers so appropriate prevention and intervention tools can be made available to the climbing community.

Consequences of DE, even without progression to a diagnosable ED, include long term negative psychological and physiological ramifications (Kärkkäinen et al., 2018). From a performance perspective, DE has been associated with unhealthy physical activity behaviors and Relative Energy Deficiency in Sport (RED-S) (Torstveit et al., 2019), a term used to describe a syndrome in active individuals who display compromised physiological functioning. The main cause is thought to be energy deficiency and may include impairments of metabolic rate, menstrual function in women, bone tissue depredation, a weakened immune system and susceptibility to injury (Mountjoy et al., 2014, 2018). A few recent studies suggest rock climbers may not be consuming adequate energy to support optimal health. These studies recognized low energy availability using 24 h dietary recall in a small group of advanced adolescent climbers (Michael et al., 2019) and 7 day food records in advanced adult climbers (Krzysztof and Judyta, 2019). Although our study did not evaluate dietary energy intake and we did not detect DE in the majority of our participants, it is possible that some of our participants, with or without DE, did not consume enough energy, which may lead to poor bone density, increased risk of injury, and compromised health (Tayne et al., 2019). It is reasonable to suggest that optimal eating behaviors which supply adequate energy and nutrients may offer protection from injury and/or help an athlete to recover from them. Further research is paramount in determining how and what rock climbers should eat to maintain health and to support performance longevity.

## Conclusion

Our study is the first to assess prevalence of DE among an international heterogeneous sample of sport lead rock climbers. Among our sample, we found a prevalence rate of 8.6%, suggesting sport lead rock climbers are not immune to DE and that the risk is elevated in female climbers (16.5%), particularly at the elite/high elite climbing ability level (42.9%).

The study had several strengths including a large sample size with an international representation of sport climbers from recreational to elite abilities. Both males and females were assessed for DE. However, it is unclear whether the percentage of climbers in each category and sex is representative of the climbing community at large. The majority of our sample was male (76.9%). Other limitations include the self-report cross-sectional nature of the study, sole focus on sport lead climbers, and need for a DE screening tool validated for use in an athletic population.

It is possible that those climbing at the highest levels may be involved in competition or maintaining/acquiring climbing sponsorships. This may lead to additional internal or external pressure to achieve a lower body weight or leanness, which ultimately could negatively affect eating patterns. Future studies should examine how the competitive climbing climate, at all skill levels, affects DE behaviors. Furthermore, studies should explore the relationship between DE and perceived importance of body weight on performance. Finally, future research should examine DE prevalence to include individuals who primarily participate in other types of climbing such as speed climbing, bouldering, traditional, and top-roping.

Our research indicates further investigations are warranted to confirm the present findings as well as how DE behaviors affect psychological and physical health and performance of adult rock climbers. It is our hope that our work will provoke further research, which includes thorough assessment of current as well as appropriate dietary patterns in these athletes. At the very least, proper education surrounding appropriate eating behavior and nutrition to support athletes in this sport is warranted, especially in female climbers.

Climbing federations can be more vocal regarding the health risks, symptoms and prevalence of DE in elite class climbers by educating coaches, trainers and athletes. Recreational and elite athletes alike, should seek professional guidance on establishing and maintaining a healthy diet appropriate to their discipline and climbing performance level.

## Data Availability Statement

The datasets generated for this study are available on request to the corresponding author.

## Ethics Statement

The studies involving human participants were reviewed and approved by Northern Michigan University's Internal Review Board #HS17-869. The patients/participants provided their informed consent to participate in this study.

## Author Contributions

LJ designed the study, collected, analyzed and interpreted the data, and drafted the manuscript. AL designed the study and edited the manuscript. GB designed the study, analyzed the data, and edited the manuscript. All authors gave final approval on the accepted manuscript.

## Conflict of Interest

The authors declare that the research was conducted in the absence of any commercial or financial relationships that could be construed as a potential conflict of interest.
